# Supplementary material for: End Sequence Analysis Toolkit (ESAT) expands the extractable information from single-cell RNA-seq data
Source: Genome Res. 2016 Oct;26(10):1397–410. doi: 10.1101/gr.207902.116 (PMC5052061; doi:10.1101/gr.207902.116)
Supplement: Supplemental Material [file supp_gr.207902.116_Supp_Materials.tar.gz › ESAT-esat_v0.1/doc/html/dir_47922fae4b2d4f7c6f8e56f6907fad50.html]

ESAT: java/umms/core Directory Reference


ESAT


- Main Page
- Related Pages
- Packages
- Classes
- Files

All Classes Namespaces Files Functions Variables Enumerator Pages

- java
- umms
- core

core Directory Reference

Directory dependency graph for core:

|  |  |
| --- | --- |
| Directories | |
| directory | alignment |
|  | |
| directory | annotation |
|  | |
| directory | coordinatesystem |
|  | |
| directory | exception |
|  | |
| directory | feature |
|  | |
| directory | general |
|  | |
| directory | model |
|  | |
| directory | readers |
|  | |
| directory | readFilters |
|  | |
| directory | scripture |
|  | |
| directory | utils |
|  | |
| directory | writers |
|  | |


---

Generated on Fri Aug 15 2014 15:11:15 for ESAT by  

 1.8.7
